# Development and validation of the questionnaire of post-pandemic coping strategies upon life return to normal for teenagers

**DOI:** 10.1192/j.eurpsy.2023.371

**Published:** 2023-07-19

**Authors:** F. Maris, E. Charmandari, A. Zartaloudi, M. Polikandrioti, C. M. Vassalos, I. Koutelekos

**Affiliations:** ^1^ National and Kapodistrian University of Athens; ^2^ University of West Attica; ^3^Greek Health System, Athens, Greece

## Abstract

**Introduction:**

COVID-19 quarantine affected teenagers’ life as it brought about significant changes in their usual way of life, disrupting every social relationships. Following the lifting of pandemic restrictions, teenagers are urged to deal with the psychological challenges of their return to normality.

**Objectives:**

To develop and validate a questionnaire in Greek teenagers to better monitor their coping strategies when returning to normal after pandemic restrictions were dropped.

**Methods:**

One hundred teenagers (41 boys, 59 girls; median age: 12) from a large provincial Greek town completed an *ab initio* 15-item questionnaire on post-pandemic coping strategies upon life return to normal for teenagers (PPCSRN-T). The responders specified their level of agreement to each item statement in five points: (1) Strongly disagree; (2) Disagree; (3) Neither agree nor disagree; (4) Agree; (5) Strongly agree. Psychometric properties were analysed. Factor analysis was performed. SPSS.21 was used for all analyses.

**Results:**

The optimal two-factor solution explained 66.1% of variance. The initial factors ‘post-pandemic daily life normalcy aspirations’ and ‘post-pandemic family life normalcy aspirations’ were reaffirmed. Item loadings were between 0.52-0.82. Each of the final factors had three items. The items ‘After pandemic restrictions are lifted, I will live an active life’, ‘After pandemic restrictions are lifted, I will make time for exercise’, ‘After pandemic restrictions are lifted, I will meet up with my friends’ represented the final factor ‘post-pandemic daily life normalcy aspirations’. The items ‘After pandemic restrictions are lifted, my family will stick to a normal daily rhythm’, ‘After pandemic restrictions are lifted, I will go on spending time with my parents’, ‘After pandemic restrictions are lifted, I will be grateful for what I will have in my life’ represented the final factor ‘post-pandemic family life normalcy aspirations’. Reliability (Cronbach alpha) for the six-item final scale was 0.62. The intra-class correlation coefficient varied from 0.50-0.73. No ceiling/floor effect was detected.

**Conclusions:**

The six-item final PPCSRN-T version proved to be a valid and reliable instrument. It would provide paediatric personnel and psychologists information on the as-yet not readily accessible coping strategies of teenagers returning to normality after the pandemic upheaval coming to an end.

**Disclosure of Interest:**

None Declared

